# Macroscale EEG characteristics in antipsychotic-naïve patients with first-episode psychosis and healthy controls

**DOI:** 10.1038/s41537-022-00329-6

**Published:** 2023-01-23

**Authors:** L. S. Dominicus, B. Oranje, W. M. Otte, K. S. Ambrosen, S. Düring, F. E. Scheepers, C. J. Stam, B. Y. Glenthøj, B. H. Ebdrup, E. van Dellen

**Affiliations:** 1 Department of Psychiatry, University Medical Center Utrecht, Utrecht, The Netherlands; 2 Center for Neuropsychiatric Schizophrenia Research (CNSR) and Center for Clinical Intervention and Neuropsychiatric Schizophrenia Research (CINS), Copenhagen University Hospital, Mental Health Center Glostrup, Glostrup, Denmark; 3 Department of Child Neurology, UMC Utrecht Brain Center, University Medical Center Utrecht, and Utrecht University, Utrecht, The Netherlands; 4 Department of Clinical Neurophysiology and MEG Center, Department of Neurology, Amsterdam Neuroscience, Vrije Universiteit Amsterdam, Amsterdam UMC, Amsterdam, The Netherlands; 5 Department of Clinical Medicine, Faculty of Health and Medical Sciences, University of Copenhagen, Copenhagen, Denmark; 6 Department of Intensive Care Medicine and University Medical Center Utrecht Brain Center, Utrecht University, Utrecht, The Netherlands

**Keywords:** Biomarkers, Psychosis, Schizophrenia

## Abstract

Electroencephalography in patients with a first episode of psychosis (FEP) may contribute to the diagnosis and treatment response prediction. Findings in the literature vary due to small sample sizes, medication effects, and variable illness duration. We studied macroscale resting-state EEG characteristics of antipsychotic naïve patients with FEP. We tested (1) for differences between FEP patients and controls, (2) if EEG could be used to classify patients as FEP, and (3) if EEG could be used to predict treatment response to antipsychotic medication. In total, we studied EEG recordings of 62 antipsychotic-naïve patients with FEP and 106 healthy controls. Spectral power, phase-based and amplitude-based functional connectivity, and macroscale network characteristics were analyzed, resulting in 60 EEG variables across four frequency bands. Positive and Negative Symptom Scale (PANSS) were assessed at baseline and 4–6 weeks follow-up after treatment with amisulpride or aripiprazole. Mann-Whitney U tests, a random forest (RF) classifier and RF regression were used for statistical analysis. Our study found that at baseline, FEP patients did not differ from controls in any of the EEG characteristics. A random forest classifier showed chance-level discrimination between patients and controls. The random forest regression explained 23% variance in positive symptom reduction after treatment in the patient group. In conclusion, in this largest antipsychotic- naïve EEG sample to date in FEP patients, we found no differences in macroscale EEG characteristics between patients with FEP and healthy controls. However, these EEG characteristics did show predictive value for positive symptom reduction following treatment with antipsychotic medication.

## Introduction

Psychosis is a syndrome defined as clinical symptoms of delusions, hallucinations, and disorganized thinking and speech^1^. Patients with a first episode of psychosis (FEP) have highly variable prognoses, where some patients only experience one psychotic episode, while others suffer from recurrent episodes or chronic symptoms of schizophrenia spectrum disorders^1^. The onset is usually during late adolescence or early adulthood and often impairs the level of functioning. Psychosis causes both societal and economic burden^2,3^, and is associated with high mortality rates including an increased risk of suicide^4,5^. In particular, diagnosis and adequate treatment of first psychosis is crucial for outcome; Reducing the time gap between the onset of a first psychotic episode and effective treatment will improve FEP patients’ prognosis^6^. Moreover, due to non-response to treatment, patients can experience more side effects, prolonged hospital admissions, and even an increased risk of suicide^6,7^. Identification of early markers of treatment response is critical to improve clinical care of patients with psychosis.

Studying antipsychotic-naïve patients can provide information about the pathophysiology of psychosis without confounding effects of treatment and the effects of chronic disease medication side effects and the use of other medication. Electroencephalography (EEG) is a low-cost and low-burden methodology which is used to characterize brain oscillations by measuring electric fields with relatively high time resolution^8^. Due to this unique temporal resolution, EEG may capture markers of complex psychotic experiences as EEG records have superior temporal resolution compared to, for example, functional-MRI^9^.

Resting state EEG (rs-EEG) is believed to reflect intrinsic activity of brain networks that is not manipulated by any form of task or stimulus presentation^10^. Rs-EEG recordings can be used to characterize the power spectrum of cortical oscillations in different frequency bands, namely delta (0.5–4 Hz), theta (4–8 Hz), alpha (8–13 Hz), beta (13–30 Hz), and gamma (>30 Hz)^11^. A decrease in alpha peak frequency and alpha power, but increases in delta and theta power have been associated with psychosis in previous studies^12–14^. Previous studies found that high alpha power was associated with poor treatment response^15–17^.

In addition, alterations in functional connectivity have been studied in patients with psychosis. This work is based on the disconnection hypothesis described in refs. ^18–20^, which states that core symptoms of schizophrenia result from dis-connectivity between distinct brain regions. EEG connectivity implicates the consideration of the relationship between two or more EEG signals^21^. Connectivity measures can be based on amplitude (e.g., amplitude envelope correlation) and phase synchronization (e.g., phase lag index). Patients as compared to controls show lower alpha phase-based connectivity, measured by (lagged) coherence and Lagged Phase Synchrony while contradicting results with other connectivity measures have been reported for higher frequency bands (beta- (13–30 Hz) and gamma- (30–200 Hz))^13^. Of interest, two studies reported that treatment response to clozapine could be predicted using EEG connectivity features with accuracies of 85% and 89.9%^22,23^. Connectivity patterns have been further characterized by higher-order measures of network organization, such as efficiency and clustering. However, studies regarding network topology in EEG found heterogeneous results related to psychosis regarding both the affected frequencies and the type of network disturbances^24–27^.

The literature on EEG in psychotic disorders shows limitations in terms of study design and methodology of EEG analysis. Studies are mainly based on psychotic populations undergoing medical treatment, while antipsychotics are known to change EEG characteristics^28,29^. Analyzing EEG characteristics in patients who are on medication naïve is, therefore, crucial to disentangle neurophysiological correlations of psychosis from medication effects. Moreover, most studies have focused on more chronic disease states of psychosis implying potentially more comorbidity, age effects on EEG, and treatment-resistant populations. Previous quantitative EEG studies of medication naïve first psychotic episode patients consisted of relatively small sample sizes (i.e., *N* = 13–31) and variable methodology (e.g., EEG recording, number of electrodes) and outcomes^25–28^. In studies on treatment response, patients received various types of antipsychotic medication with different mechanisms of action on different receptor profiles^15,30–34^. And finally, EEG characteristics used in previous studies such as coherence have shown sensitivity to errors such as spurious functional connectivity due to volume conduction)^35^. As we used state-of-the-art methodology in our analysis, differences in the definitions of EEG characteristics may have limited comparability with earlier work.

In this exploratory study, we compared rs-EEG characteristics between antipsychotic-naïve patients with a first psychotic episode and healthy controls. Based on previous studies in patients with dementia and delirium, we focus on macroscale (as opposed to regional) EEG characteristics, which showed high disease specificity^36,37^. Due to the multitude of potential characteristics of interest, heterogeneity of findings in the literature, and the scarce literature regarding antipsychotic naïve patients and EEG-based prediction models of treatment outcome, we chose a data-driven approach for variable selection. Specifically, we applied the random forest (RF) algorithm, which, along with the prediction, returns the most relevant diagnostic variables and their relative importance^38^. Thirdly, we tested if symptom reduction due to subsequent medical treatment of our FEP patients at 6-weeks follow-up could be predicted based on baseline rs-EEG characteristics using random forest regression. Based on the literature described above, we found an insufficient basis for a hypothesis-driven study to evaluate the performance of one single EEG characteristic for patient-control discrimination or treatment outcome prediction. We, therefore, used a data-driven approach to test if (either a single or a combination of) EEG characteristics could be (1) used to discriminate between patients and controls and (2) related to treatment response.

## Methods and materials

### Study population and procedure

The population studied here was recruited from three similar cohorts: The Pan European Collaboration on Antipsychotic Naïve Schizophrenia (PECANS, ClinicalTrials.gov Identifier: NCT01154829), the Pan European Collaboration on Antipsychotic Naïve Schizophrenia II (PECANSII, ClinicalTrials.gov Identifier: NCT02339844), and the OPTIMISE STUDY (ClinicalTrials.gov Identifier: NCT01555814)^39–41^. These datasets were combined because participants were included in the same center, with matching inclusion criteria. The study population consisted of two groups, patients with a first psychotic episode and healthy controls. All patients included in this study were lifetime antipsychotic naïve. Controls were matched on age, sex, and sociodemographic background. Symptoms were rated using the Positive and Negative Symptom Scale (PANSS) at baseline and at follow-up after 4–6 weeks to measure early response. Patients were treated with either amisulpride or aripiprazole according to clinical need balancing effect and side effects.

### EEG recordings

All participants underwent EEG recordings using Biosemi hardware (Amsterdam, The Netherlands) with 64 electrodes and a sample frequency of 2048 Hz. Participating subjects underwent eyes closed rs-EEG recording in the morning between 9 and 12 o’clock. EEG registrations were recorded in a quiet room (sound level <40 dB) while participants were seated in a comfortable chair and told to remain still and stay awake throughout the recording. They were asked not to smoke in the hour before the recording, nor to consume any caffeinated drinks, and were requested not to take benzodiazepines the evening before the recording from 11 pm onwards. Other medication was allowed. All resting state recordings were made after an event-related potential (ERP) recording sequence of ~45 min^42–44^.

### EEG preprocessing

**Fig. 1:**
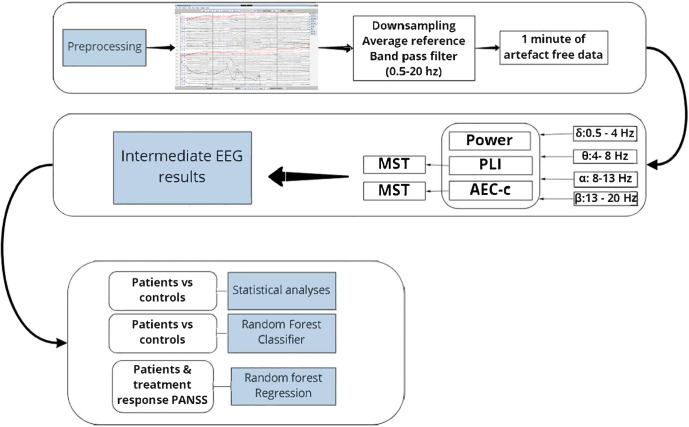
Overview of EEG processing pipeline. Raw data was downsampled and an average reference and band pass filter were applied. A total of 1 minute of combined epochs were used for analyses. For each frequency band, the power, PLI, AEC-c and MST measures were calculated. Next, we tested for differences between FEP patients and controls, (2) if EEG could be used to classify patients as FEP, and (3) if EEG could be used to predict treatment response to antipsychotic medication.

For preprocessing we used brainwave software version 0.9.152.12.26; developed by C. J. Stam, available at https://home.kpn.nl/stam7883/brainwave.html) and EEG Utils 0.6.3 in Rstudio^45^. EEG data were visually inspected for eye movement and muscle artefacts by two individual raters (L.S.D with trained students). A final check consensus meeting was applied with EvD as the final rater. An average reference was applied. Electrodes were interpolated using spherical spline if there were artefacts due to broken electrodes or other artefacts^46^. Where necessary, a maximum of six channels (~10% of the 64 channels) was accepted for interpolation, otherwise the participant was excluded from further analyzes. The first 15 epochs of 4 s without artefacts were selected. The EEG data were down-sampled to 1024 Hz to optimize the speed of data preprocessing and further analyzes for computational efficiency. For an overview of methods see Fig. 1.

### EEG characteristics

Data were band-pass filtered in four different frequency bands, which were grouped in delta (0.5–4 Hz), theta (4–8 Hz), alpha (8–13 Hz), and beta (13–20 Hz), respectively^47^. Selected features were: Relative spectral power, mean Phase Lag Index (PLI), mean Amplitude Envelope Correlation corrected (AEC-c), Minimum Spanning Tree (MST) characteristics based on PLI, and MST characteristics based on AEC-c. The feature selection of global EEG characteristics and random forest classification methodology was largely based on previous work^36,37^.

### Spectral power

The absolute spectral power was calculated using the Fast Fourier Transform. The relative power was calculated by dividing the absolute power per frequency band by the total power of the four frequency bands.

### Connectivity (AEC-c and PLI)

To calculate the functional connectivity strength we used two measures, one based on phase coupling (PLI) and one regarding amplitude coupling (AEC-c), as they appear to be complementary^48–50^.

The PLI characterizes the asymmetry in the distribution of instantaneous phase differences between two signals^48^. The PLI varies from 0 to 1, whereas 0 indicates no phase synchronization and 1 indicates complete phase locking. Zero-lag phase coupling is discarded and therefore the PLI is less sensitive to volume conduction effects and field spread.

The AEC was obtained by measuring the magnitude of the analytic signal, the analytic representation of a real-valued function. A Pearson’s correlation was calculated between the power envelopes of two signals^49^. We calculated the corrected version of the AEC (AEC-c), where signal components that pick up the same source at different EEG channels are eliminated, by performing an orthogonalization of raw signals before computing AEC values. The AEC scores range from 0 to 1, where a value of 1 indicates perfect coupling, a value of 0,5 suggests no coupling, and a value of 0 suggests perfect negative coupling^50^. The mean PLI and AEC-c were obtained for each frequency band by averaging the connectivity values of all electrode pairs.

### Network analysis

The MST was used to reconstruct a backbone of functional connections, and subsequently characterized with measures derived from graph theory. The MST is an acyclic sub-network of the brain connecting all nodes, while minimizing the link weights and reflecting most fundamental network properties^51,52^. It avoids limitations of other graph theoretical approaches such as sensitivity to connection strengths, arbitrary thresholding, or link density effects^53^. The MST was calculated based on connectivity matrices, which here represented the frequency-specific PLI or AEC-c. The connectivity matrices consisted of 64 × 64 cells (for 64 EEG channels) resulting in 64 nodes with 63 edges. All measures characterizing the topology of MST used in this study were global network measures (see Table 1).

**Table 1:** MST topology measures.

MST topology measures	Explanation
Degree (k)	Measures the number of edges/links for each node divided by the maximum number of edges possible. The maximum degree (kmax), which is the highest degree in the MST, is used for analyzes.
Leaf fraction (LF)	The ratio of leaf nodes is divided by the total number of nodes. A Leaf node (L) is a node with only one edge.
Diameter (D)	Refers to the largest distance between any two nodes. It can be interpreted as a measure of efficiency, where a low diameter indicates an efficient information flow between brain regions.
Betweenness centrality (BC)	Fraction of ll shortest paths that pass through a node. A leaf node has a BC of zero. The central node in a star-like network, is characterized by BC = 1. For the MST global measure, the highest BC (BCmax) is used.
Eccentricity (ECC)	Measure of the maximum distance calculated by the number of edges between a node and any other node in the MST. Here, we used the mean ECC of all nodes.
Tree hierarchy (Th)	Defines the hierarchy of the MST organization as optimal topology. Th is calculated as Th = *L*/ (2 M BCmax), where *L* = Leaf number and *M* = maximum leaf number.

### EEG differences between patients and controls

In initial analyses, we applied descriptive univariate EEG differences between patients and healthy controls. As these EEG features followed a non-normal distribution, a Man-Whitney *U* test was applied. Power, AEC-c, and PLI were compared in each frequency band, namely delta, theta, alpha, and beta. Next, MST features based on PLI and AEC-c, respectively, were compared in each frequency band. To correct for multiple testing the level of significance was adjusted using Holm-Bonferroni correction^54^.

### Random forest classification

In the main analyses, we applied a Random forest classifier to discriminate between FEP patients and healthy controls in R using R-statistical software version 1.4.1717, package Caret^55,56^. The model and features were a priori selected based on previous literature^36,37^. Sixty EEG characteristics were used as input information, being 15 EEG features for each frequency band, namely; Relative power, PLI, AEC-c, and twice the MST measures based on PLI or AEC-c (*k*_max_, Tree hierarchy, Diameter, leaf fraction, eccentricity, and BC_max_). For an overview see Table 2.

**Table 2:** Overview of included EEG features.

Feature name	Feature name
Delta, Relative power	Theta_MST – degree^a^
Theta, Relative power	Theta_MST – Leaf fraction^a^
Alpha, Relative power	Theta_MST – Diameter^a^
Beta, Relative power	Theta_MST – BC_max_^a^
Delta, PLI	Theta_MST – Ecc^a^
Theta, PLI	Theta_MST – Tree hierarchy^a^
Alpha, PLI	Alpha_MST – degree^a^
Beta, PLI	Alpha_MST – Leaf fraction^a^
Delta, AEC-c	Alpha_MST – Diameter^a^
Theta, AEC-C	Alpha_MST – BC_max_^a^
Alpha, AEC-C	Alpha_MST – Ecc^a^
Beta, AEC-C	Alpha_MST – Tree hierarchy^a^
Delta_MST_MST – degree^a^	Beta_MST – degree^a^
Delta_MST_Leaf fraction^a^	Beta_MST – Leaf fraction^a^
Delta_MST_Diameter^a^	Beta_MST – Diameter^a^
Delta_MST – BC_max_^a^	Beta_MST – BC_max_^a^
Delta_MST – Ecc^a^	Beta_MST – Ecc^a^
Delta_MST _Tree hierarchy^a^	Beta_MST – Tree hierarchy^a^

^a^Features are included twice, based on the PLI and AEC-c.

Random forest is a machine learning algorithm for classification and regression^38^. A random forest consists of multiple decision trees, where each tree in the forest uses a subset of the data (bootstrapping) with a subset of features. The algorithm is less prone to overfitting, reduces the variance and provides uncorrelated trees. Importantly, separate training and validation data sets are not required as the validation is built in the model itself. After multiple decision trees, the algorithm produces an accuracy of the model and variable importance scores (VIMP scores). It can be easily interpreted which features contributed most to the model. The random forest algorithm can be used for both classification and regression problems. When building a random forest, the number of decisions trees (ntree) and the number of variables calculated at each split (mtry) must be set. In our analyses, the ntree was set to 500 and the mtry was set to the square root of the number variables. The set of parameters only marginally may influence the classification outcome^57^.

The random forest classifier was applied for the classification of patients and controls based on EEG features. A tenfold cross validation was built into the model for extra internal validation. Next, a noise feature, by generating a random noise variable in R, was added to examine which features contribute to the model more than “noise”. Features containing VIMP scores lower than noise were excluded for the final model. We built different models with random subsets of the data, due to the imbalance of our data (62 patients vs 106 healthy controls). We used random subgroups (by taking random samples of 62, using R) of the controls to match the amount of patient data (*n* = 62), so the data in the model was balanced (62 patients with 62 randomly assigned healthy controls). This procedure was repeated 10 times, creating 10 different subsets of data. Subsequently, the mean accuracy, specificity and sensitivity over the subsets were calculated.

### EEG and prediction of symptom severity using random forest regression

A random forest regression model was applied to the patient group only, to investigate whether EEG features could be used to predict the reduction of symptom severity after treatment as measured by the PANSS. The same features were used as for the patients–control comparison. As the underlying pathophysiological mechanisms differ between different subscales of the PANSS, and thus likely will correspond to different biomarkers, subscales were used for analyses^58^. The following PANSS scores were used in our models; the ∆PANSS total scores, ∆PANSS positive subscale scores, ∆PANSS negative subscale scores, and ∆PANSS general subscale scores.

The ∆PANSS was calculated as:

$${\Delta}PANSS = PANSS\,follow - up-PANSS\,baseline.$$

We chose to develop tests for explained variance in absolute symptom reduction (as opposed to a percentage change in symptom severity) because antipsychotics are more effective in patients with more severe symptoms; analyzing relative improvement would therefore potentially mask predictive value^59^. Next, a permutation test was applied with 1000 permutations to the RF model. In that way, the significance of the predictive performance of the models was tested to reduce false positive results as RF regression is not straightforward for interpretation.

## Results

### Study population

A total of 62 patients and 106 matched healthy controls were included. All 60 EEG features were calculated and included for all participants. Subject characteristics are shown in Table 3. PANSS score data at 4–6 weeks follow-up were available from 45 patients. In total, 23 patients were treated with selective dopaminergic antipsychotics aripiprazole (partial D2 and 5ht1a agonist and 5HT2a antagonist), and 39 with amisulpride (D2/D3 antagonist). Dosing information was unavailable in 25 patients.

**Table 3:** Characteristics of patients and controls.

Characteristic	Patients (mean (SD))	Controls (mean (SD))
Age(years)	23.2 (4.7)	23.3(4.7)
SEX (%Male)	53%	50%
Education level (%)	1 0% 2 9.7% 3 61.3% 4 19.4%	1 4.8% 2 2.9% 3 67.3% 4 6.7%
Education of parents^a^ % highest education and income level % middle education and income level % lowest education and income level	24.2% 46.8% 22.6%	34.6% 51.0% 13.5%
Duration of illness at baseline in weeks Duration of psychosis in weeks	DUI 48.00 (62.38) DUP 106.96 (144.36)	
GAF-s Baseline (*n* = 59)	39.09 (8.19)	
GAF_F Baseline (*n* = 59)	45.56 (12.47)	
GAF-S FU weeks (*n* = 45)	56.00 (12.58)	
GAF_F FU (*n* = 45)	59.16 (12.74)	
Amisulpride mean dose (*n* = 27)^b^ Range Equivalent Olanzapine^58,59^	285 mg (170.9) 50–800 mg 7 mg	
Aripiprazole Mean dose (*n* = 9)^b^ Range Equivalent olanzapine^58,59^	10 mg (5.9) 5–20 mg 7 mg	
Co-medication at baseline Fluoxetine 20 mg Zopiclone 7.5 mg Codeine 2.5 mg	1.5%(*n* = 1) 6% (*n* = 4) 1.5%(*n* = 1)	
PANSS baseline (*N* = 60) Total Positive Negative General	74.53 (16.97) 18.57 (4.38) 18.47 (6.99) 37.50 (9.01)	
PANSS baseline of patients with follow-up data available (*N* = 45) Total Positive Negative General	71.42 (16.52) 17.91 (4.20) 17.67 (7.02) 35.84 (8.19)	
PANSS FU (*N* = 45) Total Positive Negative General	57.56 (14.10) *p* < 0.001* 12.73 (3.77) *p* < 0.001* 16.42 (6.05) *P* = 0.17 28.40 (7.32) *P* < 0.001*	

*Significant result, *p* values < 0.05.

^a^1: Education level: University or similar; 2: bachelor or similar/skilled worker; 3 currently active in education; 4 no education.

^b^Incomplete datasets due to missing corresponding dose.

### EEG differences between patients and controls

After correction for multiple testing, none of the comparisons between groups showed significant differences. For comparisons with previous literature, we report comparisons with uncorrected *p* values < 0.05. The average delta relative power was higher for patients *(Median (M)* = 0.659) compared to controls *(M* = 0. 0.575*; uncorrected p* = 0.044).

**Fig. 2:**
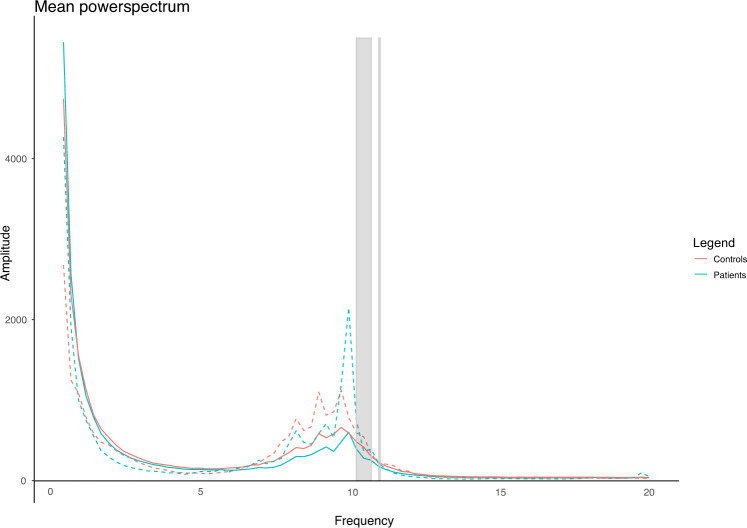
Mean power spectra with standard deviation of patients and controls. The gray vertical bar represents significantly different bins between the two groups (*p* < 0.05, uncorrected for multiple testing).

**Fig. 3:**
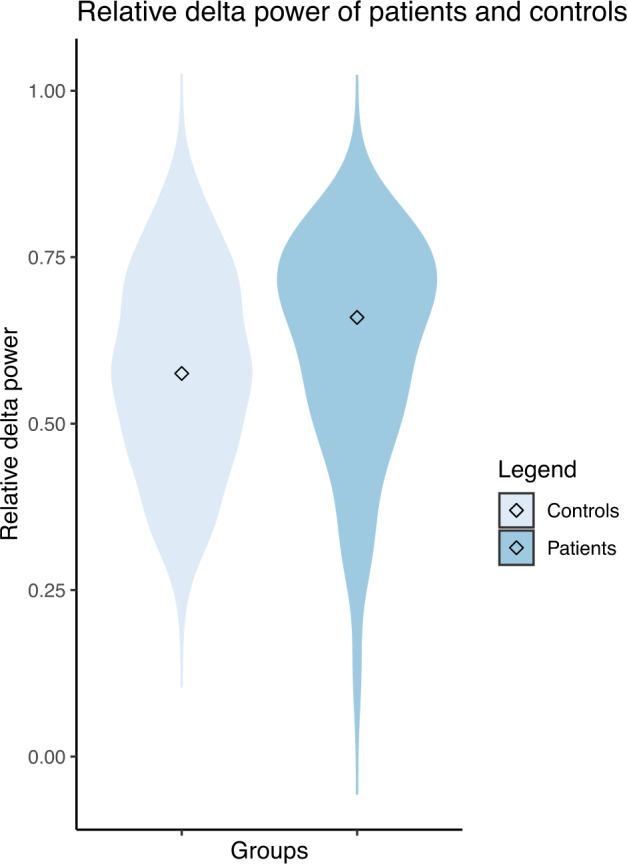
Violin plots of the relative delta power and ECC with median shown inside for both controls and patients. The distribution of the values (Relative delta power and ECC) is shown by the shape of the plot.

**Fig. 4:**
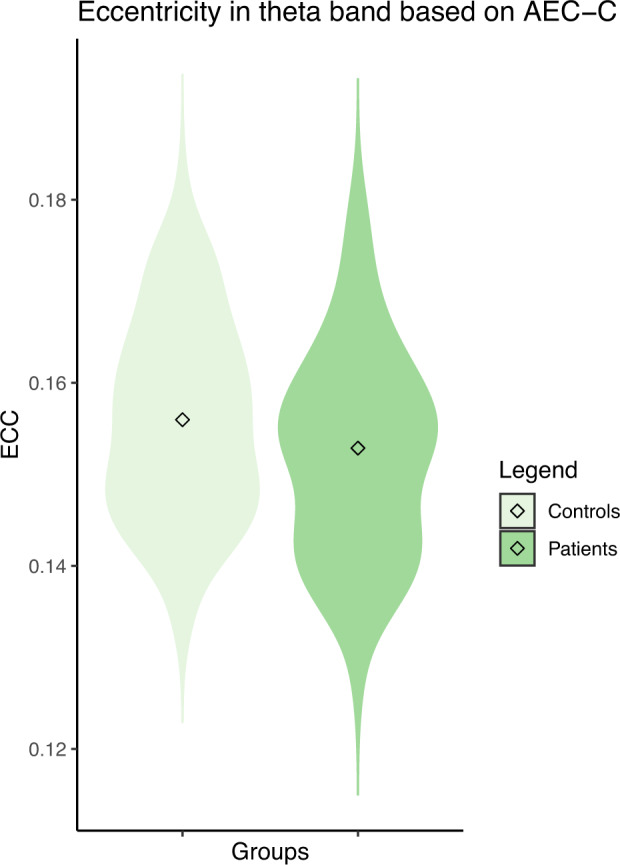
Violin plots of the relative delta power and ECC with median shown inside for both controls and patients. The distribution of the values (Relative delta power and ECC) is shown by the shape of the plot.

Absolute power per frequency bin was also compared between the two groups, to identify subtle group differences in the power spectrum that may be masked by averaging the power in broader frequency ranges (Fig 2). The absolute power in 10.25–10.5, 10.50–10.75, and 11.00–11.25 Hz was lower in patients compared to controls (*p* = 0.044, *p* = *0.029, and p* = 0.049*, respectively, uncorrected for multiple testing*). Based on the theta band AEC-c, the Eccentricity in patients was lower *(M* = 0.153 versus 0.156*;* uncorrected *p* = 0.041*)*. Results are shown in Figs. 3 and 4.

Finally, exploratory analyses showed no differences between severely ill and treatment-responsive patients compared to controls for any of the characteristics (see Supplement S6).

### Random forest classifier

Application of the random forest classifier to classify patients and controls showed that 40 out of 60 EEG features performed better than a random noise feature, leading to a mTry of 6 (*mTry* = *square root of the number variables)*. The random forest classification resulted in a mean accuracy of 50.2% for the differentiation between patients and controls. The mean sensitivity and specificity scores were 52.2% and 48.5%, respectively.

### Random forest regression

**Fig. 5:**
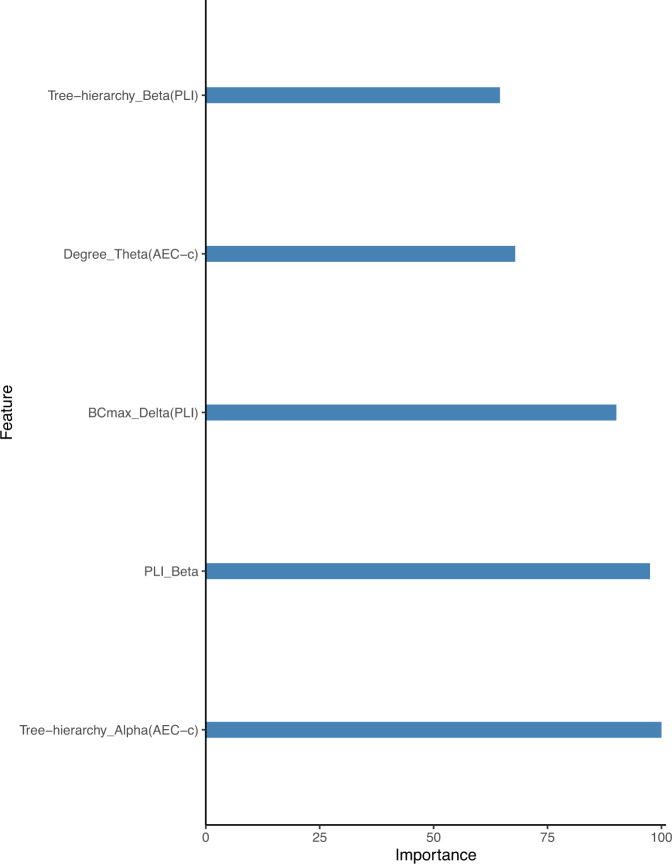
Relative importance scores of the five best features in the random forest regression when predicting delta PANSS positive. Feature scores can vary from 0 to 100, where 100 means the highest feature importance.

All 45 patients with available baseline and follow-up PANSS scores were included in the prediction of treatment response. Again, 60 features were included in the model (Table S1). Ntree was set to 500 and mTry to 8 (*mtry* = *square root of the number variables*). Explained variance above change level was found for the regression model with the outcome ∆PANSS positive at 4–6 weeks (*R*² = 0.23, *p* = 0.004). Results are shown in Table 4 and feature importance is shown in Fig. 5. The most important features were the Th in the alpha band (AEC-c), PLI in the beta band, and the BCmax in the delta band (PLI). Scatterplots of best individual features are shown in Supplement Fig. S3. Exploratory analysis on random forest classification or regression with individual PANSS as outcomes instead of total PANSS scores showed no significant findings; dividing the groups in medication cohorts (amisulpride and aripiprazole) for analyses also showed no significant results.

**Table 4:** Regression scores for predicting treatment response after 6 weeks of treatment.

Absolute PANSS (Follow-up – Baseline)	RMSE	*R*^2^	*P* value
∆Total PANSS score	14.19(1.64)	0.09(0.11)	0.248
∆Positive PANSS score	3.78(0.61)	0.23(0.14)	*0.004
∆Negative PANSS score	6.70(0.71)	0.04(0.05)	0.675
∆General PANSS score	7.51(1.29)	0.06(0.07)	0.743

Provided are the root mean square error (RMSE), where a lower RMSE means a better fit to the model and the R squared, where a R squared close to 1 means a strong relationship. If R squared is 1, the model accounts for 100% variation. *P < 0.05.

## Discussion

In this exploratory study with the largest sample (*n* = 62) of antipsychotic-naïve FEP patients to date, we found no differences in quantitative EEG characteristics between FEP patients and healthy controls. Using these EEG characteristics as input to a random forest classifier in all 45 patients with available PANSS scores, our model showed chance level discrimination between patients and controls. Nevertheless, a RF regression based on baseline EEG characteristics did explain 23% variance in positive symptoms reduction after 4–6 weeks of treatment with antipsychotic medication.

Our results point towards a contribution of EEG features in predicting efficacy of antipsychotic treatments for positive symptoms in psychosis. This is in line with previous reports on clozapine treatment response prediction in chronic patients with schizophrenia using EEG features, resulting in high (85–89.9%) accuracies^22,23^. To the best of our knowledge, our current study is the first to use a random forest regression model to predict treatment response in FEP patients. The features that showed most contribution to the prediction of positive symptoms were Tree hierarchy (alpha band, AEC-c), PLI (beta), maximum Degree (Theta, AEC-c), Tree hierarchy (beta band, PLI) and maximum Betweenness centrality (delta band, PLI). A larger reduction in PANSS positive scores appeared associated with higher PLI and Tree hierarchy (PLI) in the beta band, lower degree in the theta band (AEC-c), and tree hierarchy in the alpha band (AEC-c) at baseline (see Supplement S5). These results imply that network characteristics, specifically centrality and hierarchy characteristics in multiple frequency bands, may be used as predictor of treatment response in FEP. As the effects were found in different frequency bands and based on two different connectivity measures, we remain cautious in the physiological interpretation of this finding. A challenge for future work is to replicate these results using a simpler metric of EEG network organization.

It is noteworthy that we found no rs-EEG differences between patients and controls, but our EEG-based random forest regression did significantly explain variance in positive symptoms in patients. An explanation might be that patients with severe symptoms at baseline show deviations in their rs-EEG related to treatment response, which is masked in group-level comparisons to controls when all patients are included in the analysis. Moreover, explorative analyses indicated no differences between severely ill and treatment-responsive patients compared to controls (see Supplement S6).

Previous studies also reported EEG abnormalities in patients with schizophrenia compared to controls. Without correction for multiple testing, our results showed an increased delta power which is in line with previous literature^12–14^. Previous work on network differences between patients with schizophrenia and controls has been inconsistent, and the trend of theta band ECC differences found in our study has not been described before^25,27^.

We could not replicate previous findings regarding classification of patients with schizophrenia and healthy controls based on EEG features^60–62^. Possible limitations in previous studies were medication effects and duration of disease. A longer disease duration may induce EEG alterations on macroscale EEG characteristics that are not yet observed in FEP. Previous studies were also based on relatively small sample sizes.

### Strengths and limitations

We analyzed a relatively large EEG dataset of antipsychotic-naïve FEP patients, using both conventional statistical group comparisons and a data-driven approach. The use of different types of EEG features, namely spectral power, connectivity, and network topology features made it possible to study relevant combinations of EEG variables.

We used PANSS scores as continuous outcome variable instead of dichotomization of treatment response. The definition of treatment response is an ongoing point of discussion. Andreasen and others developed criteria to define remission in patients with a first psychotic episode^63^. These criteria consist of an improvement of several scores of the PANSS over 6 months and is therefore quite strict and might be a limitation to classify patients with a first psychotic episode into remission after 4–6 weeks. To use a cut-off of a percentage reduction in PANSS scores is a frequently used alternative, but lack of consensus exists in the literature on the cut-off value of choice^64–66^. We, therefore, considered a continuous outcome measure of treatment response more appropriate. Lastly, as RF regression models are not straightforward to interpret, we used permutation tests to the model to reduce the chance of false positive findings.

A limitation of this study is that we could not compare outcomes to a placebo condition and our regression analysis was not externally validated in another dataset, which may limit the generalizability of findings. Next, patients in our study were either treated with dosages of amisulpride or aripiprazole, that were converted to a Daily Defined Dose of olanzapine^67,68^. Of note, the mean doses were lower than the recommended minimally effective treatment dose in studies of treatment resistance in 50% of the patients^60,61,69^. It cannot be excluded that a subgroup of patients might have responded to a higher dose. However, high-dose treatment may have induced intolerable levels of side effects which in turn may have compromised study retention, since it is well-known that antipsychotic-naïve patients are much more sensitive to the development of unwanted side effects of antipsychotics than are more chronic patients^70^.

In our study, we analyzed up to 20 Hz and excluded higher frequencies, because previous literature found that signals with frequencies above 20 Hz are contaminated with muscle activity^47,71^. Nevertheless, previous literature that included gamma bands for analyses found increased gamma power in antipsychotic-treated FEP patients compared to controls and increased gamma connectivity in patients with schizophrenia, but these findings may be confounded by e.g., extrapyramidal side effects^72,73^.

The rs-EEG data were obtained after patients underwent task-related EEG recordings including PPI, P50 suppression, mismatch negativity, and selective attention paradigms. The resting state recording was only performed after patients had completed the tasks and were able to do so, which might have caused a selection bias towards better-performing subjects in our dataset.

Regarding our methods, we a priori set an arbitrary cut-off point of six channels for interpolations, otherwise the participant was excluded for further analyses. Based on this criterion, one patient and four controls were excluded (see Supplmentary S7). A different cut-off could have led to slightly different findings.

We opted for a random forest classification and regression model, nevertheless, other machine learning models could also be applied. Before our model would be clinically applicable, external validation need to be performed on a different data set with other EEG equipment. In that way, the potential generalizability of our results can be ensured.

Our analyses were limited to macroscale, whole-brain EEG characteristics. The selection of other features or regional analysis (in source-space) may lead to other findings and is subject to further studies. Similarly, deep learning analysis of raw or minimally preprocessed EEG signals might be a solution to avoid pre-selection of features^74^.

The main objective of our current study was to develop a solid EEG model for predicting treatment efficacy and the potential added value of clinical models can only then be properly investigated. However, the use of a prediction model containing both EEG characteristics and clinical information might increase the performance of the model^1^. Adding other EEG features or measures based on completely different methodologies, i.e., PET (f)MRI might also be of interest for future research.

## Conclusion

In conclusion, our results suggest predictive value in macroscale quantitative EEG characteristics for antipsychotic treatment response regarding positive symptoms in antipsychotic-naïve, first-episode patients with psychosis. Previous findings on rs-EEG in patients with psychotic disorders in comparison to control subjects may be influenced by medication effects and/or (primary and secondary) effects of ongoing psychotic illness.

## Supplementary information

Supplements
