# Supplementary material for: Macroscale EEG characteristics in antipsychotic-naïve patients with first-episode psychosis and healthy controls
Source: Schizophrenia (Heidelb). 2023 Jan 23;9(1):5. doi: 10.1038/s41537-022-00329-6 (PMC9870995; doi:10.1038/s41537-022-00329-6)
Supplement: Supplementary file 1 — Supplements [file 41537_2022_329_MOESM1_ESM.pdf]

# Supplements

<u>Content:</u>

Supplement S1: Overview inclusion criteria studies

Supplement S2: Outcome of RF classifier subsets

Supplement S3: Mean importance scores of random forest classifier

Supplement S4: Importance scores of random forest regression (PANSSpositive)

Supplement S5: Scatterplots of best features random forest regression (PANSSpositive)

Supplement S6: Explorative analyses

Supplement S7: Data exclusion and interpolation

# Supplement S1

**OPTIMISE STUDY**

**Inclusion Criteria:**

- Schizophrenia, schizophreniform or schizoaffective disorder (DSM-IV)
- Age 18-40 years
- Written informed consent.

**Exclusion Criteria:**

- A time interval between the onset of positive symptoms (hallucinations and/or delusions) and study entry exceeding two years.
- Prior use of antipsychotic medication longer than an episode of two weeks in the previous year and/or 6 weeks lifetime. However, for this study patients who had used antipsychotics in the past were excluded.
- Intolerance to one of the drugs in this study. Patients who are coercively treated at a psychiatric ward (based on a judicial ruling)
- Patients who are represented by a legal ward or under legal custody
- The presence of one or more of the contraindications against any of the study drugs as mentioned in the SPC texts
- Pregnancy, as determined through a pregnancy test, or lactation

**PECANS I STUDY**

**Inclusion Criteria:**

- Fulfilling the diagnostic criteria of schizophrenia or schizoaffective disorder according to ICD-10 (International Classification of Diseases version 10) or DSM-IV/V (Diagnostic and Statistical Manual version 4 /5)
- Age 18-45 years
- Never treated with antipsychotic compounds or central nervous system (CNS) stimulants
- Legally competent

**Inclusion criteria controls:**

- Matching patients on age (+/- 2 years), sex and parental socioeconomic status
- Age 18-45 years
- No psychiatric or physical disease

**Exclusion Criteria:**

Patients: mental retardation, other chronic diseases, use of ant depressive medicine during the last month, being pregnant, ongoing substance abuse

Controls: psychiatric diagnosis, psychiatric diagnosis in first-degree relatives, drug abuse, mental retardation

**PECANS II STUDY**

**Inclusion Criteria Patients:**

- Fulfilling the diagnostic criteria of schizophrenia or schizoaffective disorder according to ICD-10 (International Classification of Diseases version 10) or DSM-IV/V (Diagnostic and Statistical Manual version 4 /5)
- Age 18-45 years
- Never treated with antipsychotic compounds or central nervous system (CNS) stimulants
- Legally competent

**Inclusion criteria controls:**

- Matching patients on age (+/- 2 years), sex and parental socioeconomic status
- Age 18-45 years
- No psychiatric or physical disease

Exclusion Criteria patients**:**

- Substance abuse during the last 3 month or patients fulfilling the criteria of ongoing substance abuse due to ICD-10/DSM-IV/V
- Treatment with antidepressant during the last 30 days
- Head injury with more than 5 minutes of unconsciousness
- Patients involuntarily admitted or treated
- Components of metal implanted by operation
- Pacemaker
- Pregnancy
- Severe physical illness

- Exclusion criteria controls
- First degree relatives with psychiatric disease
- Substance abuse during the last 3 month or positive screening of drugs in urine-sample
- Head injury with more than 5 minutes of unconsciousness
- Components of metal implanted by operation
- Pacemaker
- Pregnancy
- Severe physical illness

## Supplement S2

| **Group and feature selection** | **Accuracy (%)** | **Sensitivity (%)** | **Specificity (%)** |
|---|---|---|---|
| Subset 1 | 49,5 | 54,7 | 44,3 |
| Subset 2 | 50,1 | 49,9 | 52,2 |
| Subset 3 | 48,3 | 47,7 | 49,0 |
| Subset 4 | 51,4 | 60,6 | 42,3 |
| Subset 5 | 48,8 | 52,2 | 45,4 |
| Subset 6 | 52,1 | 50,1 | 53,7 |
| Subset 7 | 49,7 | 53,6 | 46,0 |
| Subset 8 | 49,3 | 49,3 | 49,4 |
| Subset 9 | 50,0 | 49,1 | 51,0 |
| Subset 10 | 53,0 | 54,7 | 51,3 |
| **Mean** | **50,2** | **52,2** | **48,5** |

Table S1. Outcome of random forest classifier different subsets. ntree = 500, mtry = 6

# Supplement S3

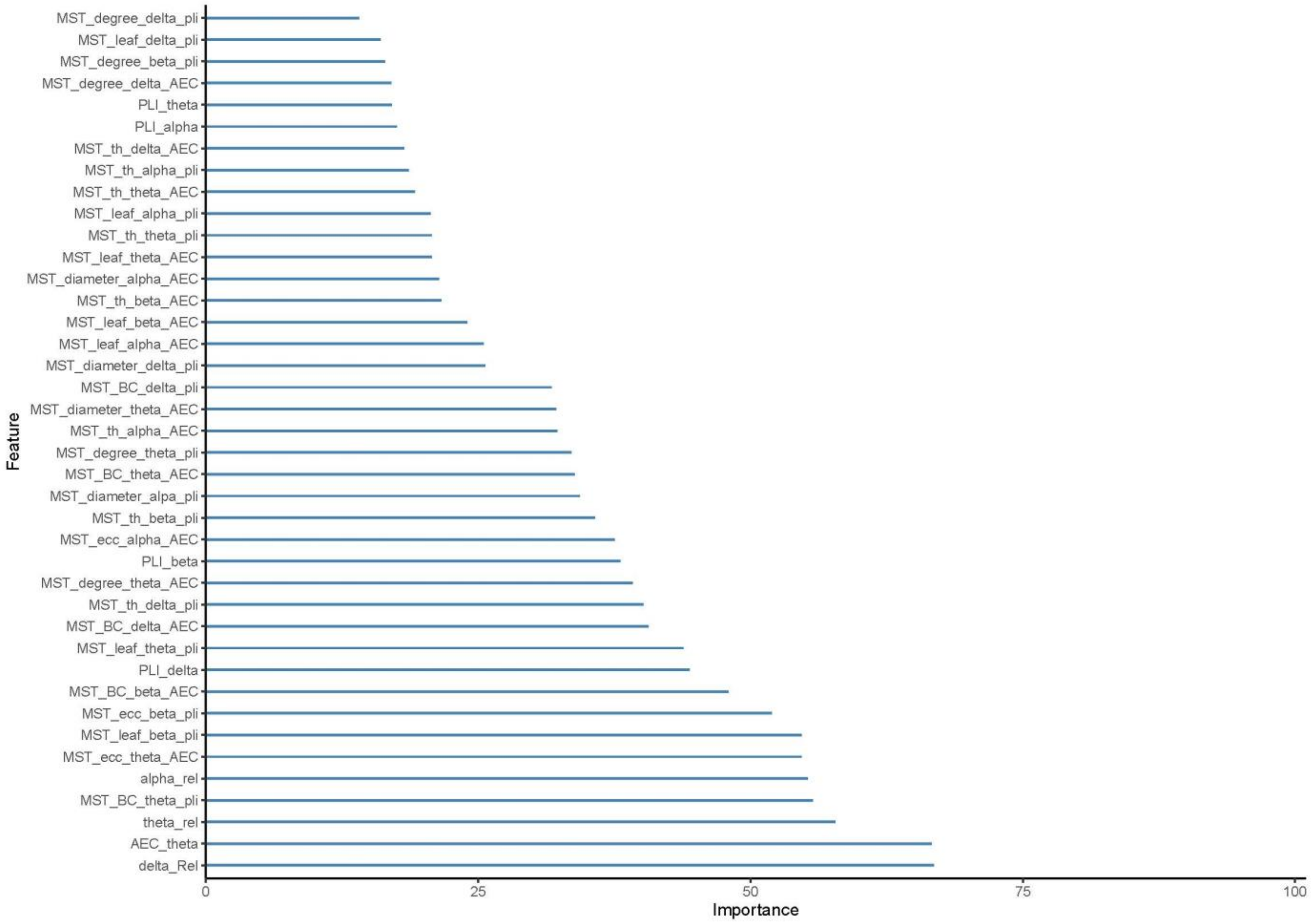


**Figure S1.** Mean relative variable importance scores for random forest classifier discriminating between patients and controls. Scores range from 0 to 100, meaning the relative delta power has relatively the best importance score compared to the other variables. Only variables with more contribution than a random noise feature are shown.

Excluded features after adding a random noise feature were; AEC-c alpha, AEC-c beta, relative alpha power, relative delta power, relative theta power, BC alpha (AEC-c), BC beta (AEC-c), BC theta, (PLI), Degree alpha (PLI), Degree alpha (AEC-c), Diameter alpha (AEC-c), Diameter theta (AEC-c), diameter beta (PLI), Diameter beta (AEC-c), ECC alpha (PLI), ECC delta AEC-c), ECC alpha (AEC-c), ECC theta (AEC-c), ECC beta (PLI), Leaf fraction beta (PLI)

# Supplement S4

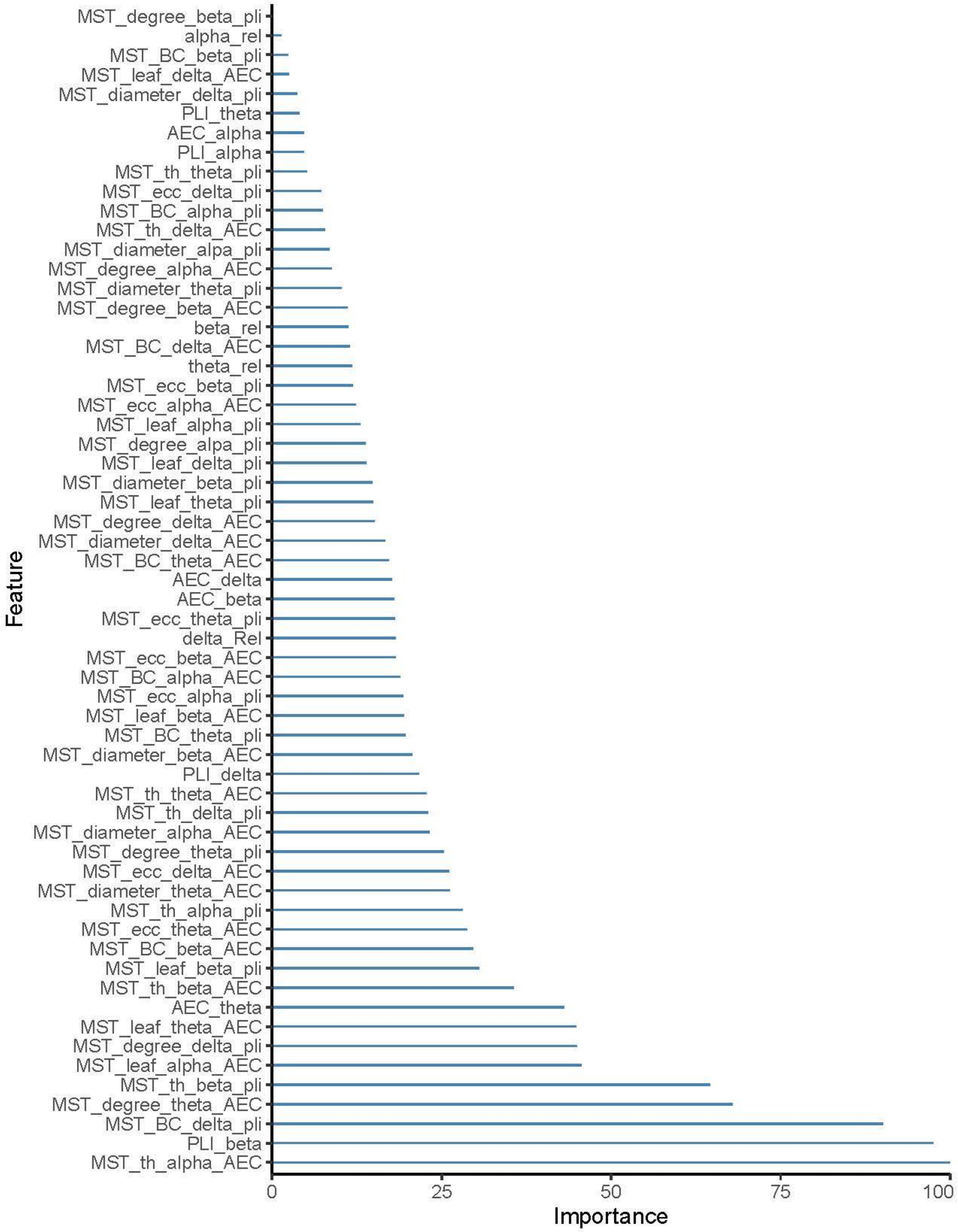


**Figure S2.** Mean relative variable importance scores for random forest regression and PANSS positive scores of all variables. Scores range from 0 to 100, meaning the Tree hierarcy (Th) in the alpha band based on the AEC-c has relatively the best importance score compared to the other variables.

# Supplement S5

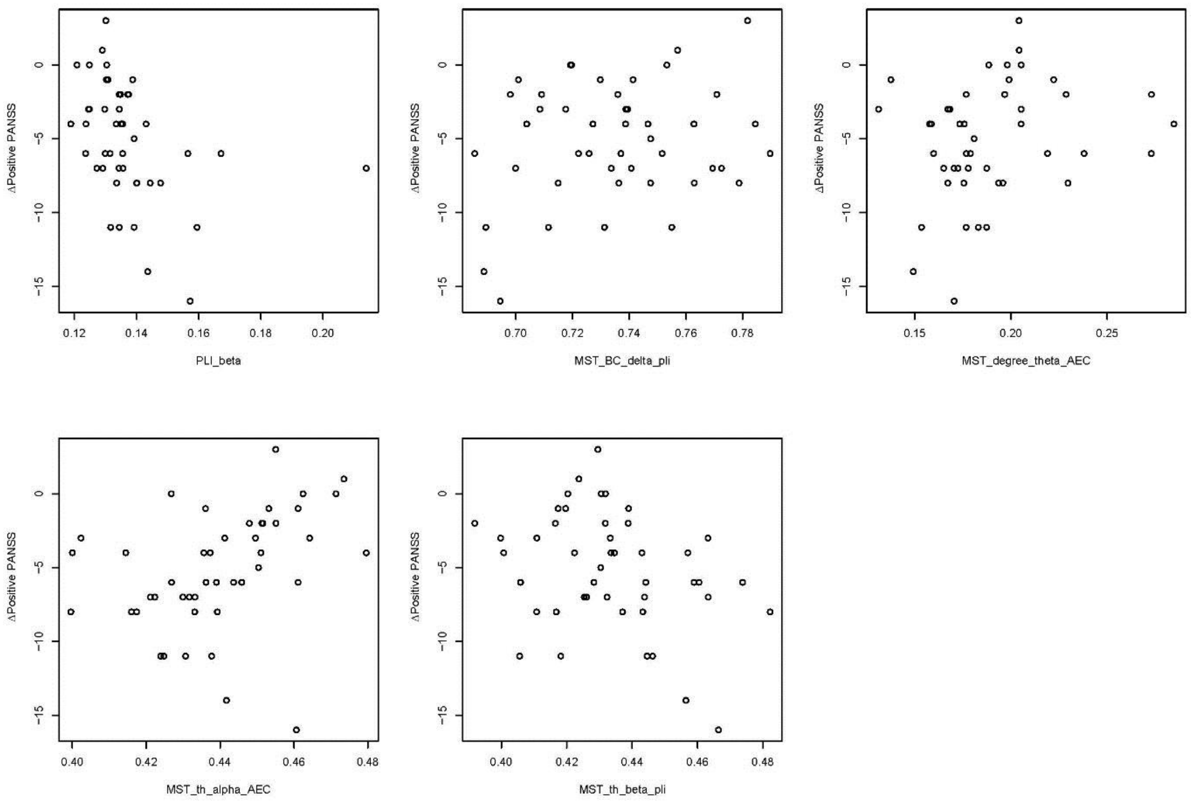


**Figure S3** Scatter plots of the 5 best features for the random forest regression model with PANSS positive scores. On the Y axis the Δpositive PANSS positive scores and on the X axis the feature name.

## Supplement S6

Explorative analyses

To identify the reason there were no differences between patients and healthy controls, which was in contrast to the result that PANSS positive showed a predictive value, we applied some explorative analyses.

The ten patients with the highest ( meaning most symptoms) PANSS positive baseline scores were identified and compared to healthy controls. Only the EEG features that contributed most to the model of regression were compared between the two groups. There were no significant differences found between the two groups.

| Feature | Test | P Value |
|---|---|---|
| Tree hierarchy alpha (AEC-c) | Wilcoxon rank sum test | 0.155 |
| Bcmax delta (PLI) | Two sample T test | 0.971 |
| Tree hierarchy beta (PLI) | Two sample T test | 0.074 |
| PLI beta | Wilcoxon rank sum test | 0.114 |
| Degree theta (AEC_c) | Wilcoxon rank sum test | 0.764 |

Table S4 Explorative analyses *$p<0.05$

## Supplement S7

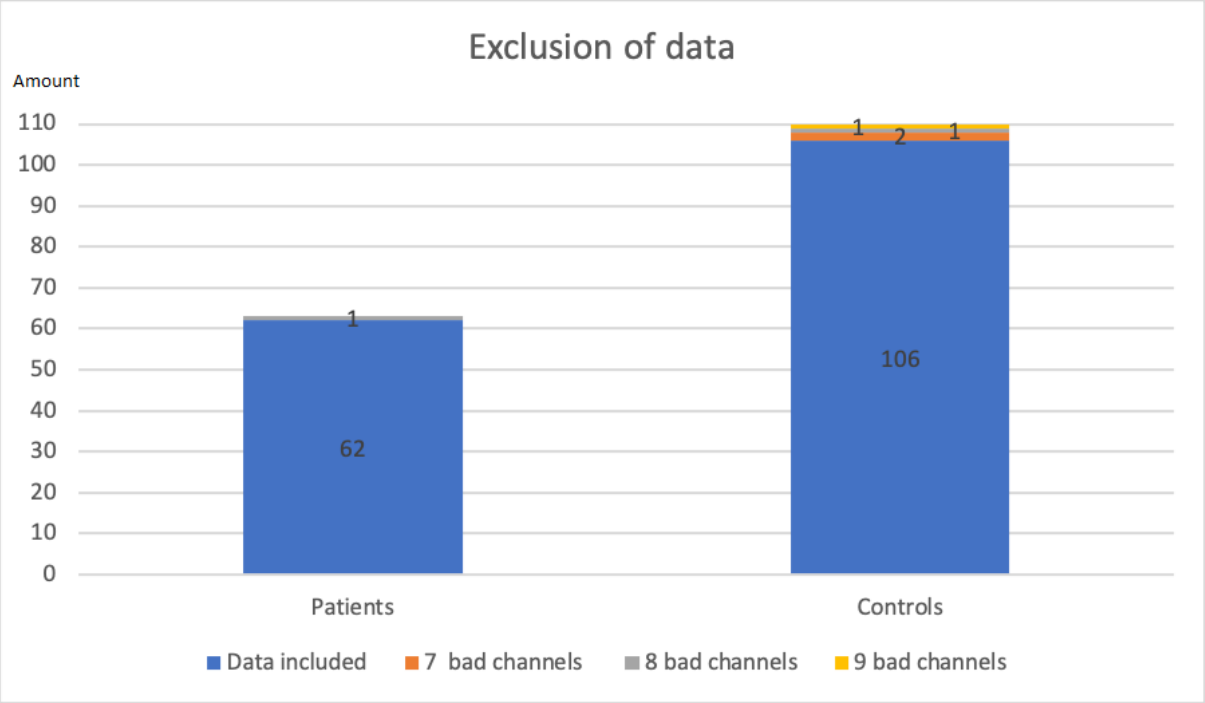


**Figure S4** Exclusion of data due to bad channels. One patient and four controls were excluded for exceeding the amount of more than six channels that required interpolation.

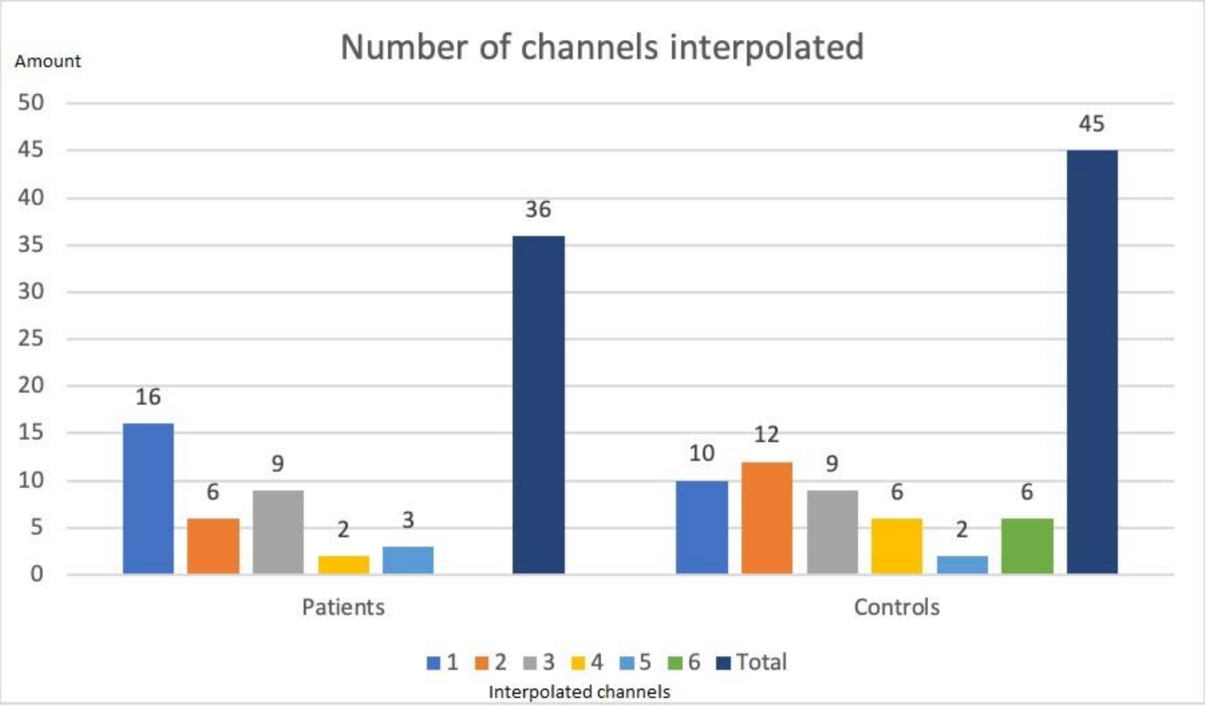


**Figure S5** Overview of number of interpolated channels of the included data for both patients and controls. On the y-axis the amount of patients/controls and on the x-axis the different number of interpolated channels.
